# Effect of norfloxacin and moxifloxacin on melanin synthesis and antioxidant enzymes activity in normal human melanocytes

**DOI:** 10.1007/s11010-014-2297-7

**Published:** 2014-11-30

**Authors:** Artur Beberok, Dorota Wrześniok, Michał Otręba, Maciej Miliński, Jakub Rok, Ewa Buszman

**Affiliations:** Department of Pharmaceutical Chemistry, Faculty of Pharmacy, Medical University of Silesia, Jagiellońska 4, 41-200 Sosnowiec, Poland

**Keywords:** Norfloxacin, Moxifloxacin, Melanin, Tyrosinase activity, Oxidative stress

## Abstract

Fluoroquinolone antibiotics provide broad-spectrum coverage for a number of infectious diseases, including respiratory as well as urinary tract infections. One of the important adverse effects of these drugs is phototoxicity which introduces a serious limitation to their use. To gain insight the molecular mechanisms underlying the fluoroquinolones-induced phototoxic side effects, the impact of two fluoroquinolone derivatives with different phototoxic potential, norfloxacin and moxifloxacin, on melanogenesis and antioxidant enzymes activity in normal human melanocytes HEMa-LP was determined. Both drugs induced concentration-dependent loss in melanocytes viability. The value of EC_50_ for these drugs was found to be 0.5 mM. Norfloxacin and moxifloxacin suppressed melanin biosynthesis; antibiotics were shown to inhibit cellular tyrosinase activity and to reduce melanin content in melanocytes. When comparing the both analyzed fluoroquinolones, it was observed that norfloxacin possesses greater inhibitory effect on tyrosinase activity in melanocytes than moxifloxacin. The extent of oxidative stress in cells was assessed by measuring the activity of antioxidant enzymes: SOD, CAT, and GPx. It was observed that norfloxacin caused higher depletion of antioxidant status in melanocytes when compared with moxifloxacin. The obtained results give a new insight into the mechanisms of fluoroquinolones toxicity directed to pigmented tissues. Moreover, the presented differences in modulation of biochemical processes in melanocytes may be an explanation for various phototoxic activities of the analyzed fluoroquinolone derivatives in vivo.

## Introduction

Fluoroquinolones constitute a relatively new class of antibiotics since they were introduced to therapy in 1980. These antibiotics display a broad spectrum of antibacterial activity including strong effects on Gram-negative aerobic and anaerobic organisms as well as on Gram-positive and atypical pathogens [1, 2]. The mechanism of action of fluoroquinolones involves inhibition of deoxyribonucleic acid (DNA) gyrase (topoisomerase II) and topoisomerase IV, enzymes involved in bacterial DNA replication, transcription, repair, and recombination [3, 4]. Norfloxacin, with potent activity against urinary pathogens, is used for the treatment of common as well as complicated urinary tract infections, whereas moxifloxacin is approved and considered as an alternative to β-lactams and macrolides for the treatment of acute bacterial sinusitis and lower respiratory tract infections [2, 5]. Despite their excellent antibacterial properties, fluoroquinolones cause phototoxic reactions as one of the main side effects, what introduces a serious limitation to their use. Literature data report many examples of fluoroquinolones-mediated skin phototoxicity. These reactions include skin lesions with different degree of severity, from cutaneous reactions (erythema, rashes, toxic dermatitis, Steven-Johnson syndrome, or toxic epidermal necrolysis) to skin cancer (carcinoma and melanoma) [6–11]. The photoreactivity of fluoroquionolones is markedly modulated by the nature and position of the substituents attached to the quinolone’s structure. These antibiotics undergo a variety of photochemical processes such as dehalogenation, oxidation of an amino substituent at C-7, decarboxylation, and the formation of reactive oxygen species (ROS), such as hydroxyl radical, superoxide anion, hydrogen peroxide (Type I), and singlet oxygen (Type II). These processes are able to modify cell components, including lipids, proteins, and nucleic acids [3, 8]. Halogenation (chlorine, fluorine) of position 8 together with fluorination of position 6 (the so-called double-halogenated quinolones) has demonstrated significant phototoxic potential [2, 8]. Therefore, lomefloxacin and sparfloxacin have been reported to have relatively high phototoxic potential as compared with other fluoroquinolones, e.g., ciprofloxacin or norfloxacin which are capable to induce severe phototoxic reactions when the patient is exposed to high drug dosages and high levels of UV radiation. [3, 8, 12]. Moxifloxacin is a new fluoroquinolone antibiotic with a methoxyl group at position 8 of the quinolone system what confers photostability and reduced phototoxicity on the molecule [3, 6, 8].

Melanocytes form a heterogeneous group of cells in human body. Although all of them have ability to produce melanin, their functions in all target places are much wider than only the melanin synthesis. They protect epidermal cells from damage by limiting the penetration of UV rays through the epidermal layers and scavenging reactive oxygen species generated in response to UV exposure. Moreover, melanocytes are also phagocytic cells involved in the inflammatory response [13–15]. In the human body, melanocytes are present not only in the epidermis, hair, and iris where they give a color to these structures but also in the inner ear, central nervous system, and heart [13, 15].

Epidermal melanin can be divided into two classes: eumelanin with its known photoprotective properties and pheomelanin which is presumed to be phototoxic [14, 16]. Moreover, melanin has an affinity for drugs and other chemical substances. Because of these properties, melanin efficiently filters toxic substances and protects tissues from oxidative and chemical stress. However, with chronic exposure to toxic substances, the properties of melanin change so that under severe oxidative stress and binding of excessive amount of toxins, melanin itself may induce damage to cells [17]. In addition, drug bound to melanin forms a depot that releases the drug over a long period and increases the level of noxious substances stored in melanin, what may cause degeneration in melanin-containing cells (especially in the skin or eye) and surrounding tissues [18].

Previously, we have documented that ciprofloxacin [19] and lomefloxacin [20] modulate biochemical processes in normal human melanocytes, suggesting a mechanism for the drug-induced toxicity on pigmented tissues. Whether the same mechanism can be attributed to other fluoroquinolone derivatives, i.e., norfloxacin and moxifloxacin, with different toxic potential, remains to be determined.

The aim of the present work was to examine the effect of norfloxacin and moxifloxacin on melanogenesis and antioxidant defense system in cultured normal human melanocytes HEMa-LP.

## Materials and methods

### Materials

Norfloxacin, phosphated-buffered saline (PBS), 3,4-dihydroxy-l-phenylalanine (L-DOPA) and amphotericin B were purchased from Sigma-Aldrich Inc.(USA). Moxifloxacin hydrochloride (Avelox^TM^ solution for *i.v.* use containing 400 mg of moxifloxacin in 0.8 % saline) was obtained from Bayer Healthcare Pharmaceuticals Inc. (Germany). Neomycin sulfate was obtained from Amara (Poland). Penicillin was acquired from Polfa Tarchomin (Poland). Growth medium M-254 and human melanocyte growth supplement-2 (HMGS-2) were obtained from Cascade Biologics (UK). Trypsin/EDTA was obtained from Cytogen (Poland). Cell Proliferation Reagent WST-1 was purchased from Roche GmbH (Germany). The remaining chemicals were produced by POCH S.A. (Poland).

### Cell culture

The normal human epidermal melanocytes HEMa-LP (Cascade Biologics, UK) were grown according to the manufacturer’s instruction. The cells were cultured in M-254 basal medium supplemented with HMGS-2, penicillin (100 U/ml), neomycin (10 μg/ml,) and amphotericin B (0.25 μg/ml) at 37 °C in 5 % CO_2_. All experiments were performed using cells in the passages 6-9.

### Cell viability assay

The viability of melanocytes was evaluated by the WST-1 (4-[3-(4-iodophenyl)-2-(4-nitrophenyl)-2H-5-tetrazolio]-1,3-benzene disulphonate) colorimetric assay according to the method described earlier [19, 21].

### Measurement of melanin content

The melanocytes were seeded in T-25 flasks at a density of 1 × 10^5^ cells per flask. Norfloxacin or moxifloxacin treatment in a concentration range from 0.005 to 0.5 mM began 48 h after seeding. After 24 h of incubation, the cells were washed three times with PBS and detached with trypsin–EDTA. Cell pellets were placed into Eppendorf tubes, dissolved in 100 μl of 1 M NaOH at 80 °C for 1 h, and then centrifuged for 20 min at 16,000×*g*. The supernatants were placed into a 96-well microplate, and absorbance was measured at 405 nm—a wavelength at which melanin absorbs light [22]. A standard synthetic melanin curve (0–400 μg/ml) was performed in triplicate for each experiment. Melanin content in norfloxacin and moxifloxacin-treated cells was expressed as the percentage of the controls (untreated melanocytes).

### Tyrosinase activity assay

Tyrosinase activity in HEMa-LP cells was determined by measuring the rate of oxidation of L-DOPA to DOPAchrome, according to the method described by Kim et al. [23] and Busca et al. [24], with a slight modification. The cells were cultured at a density of 1x10^5^ cells in T-25 flasks for 48 h. After 24-h incubation with norfloxacin or moxifloxacin (concentration range from 0.005 to 0.5 mM), cells were washed three times with PBS, lysed, and clarified by centrifugation at 10,000×*g* for 5 min. A tyrosinase substrate L-DOPA (2 mg/ml) was prepared in the same lysis phosphate buffer. 100 μl of each lysate were put in a 96-well plate, and the enzymatic assay was initiated by the addition of 40 μl of L-DOPA solution at 37 °C. Absorbance was measured every 10 min for at least 1.5 h at 475 nm using a microplate reader. Tyrosinase activity was expressed as the percentage of the controls.

A cell-free assay system was used to test for direct effects on tyrosinase activity. 130 μl of phosphate buffer containing norfloxacin or moxifloxacin in a concentration range from 0.005 to 1.0 mmol/l were mixed with 20 μl of mushroom tyrosinase (1,000 units), and 100 μl of L-DOPA solution (2 mg/ml) was added to each well. The assay mixtures were incubated at 37 °C for 20 min, and absorbance of DOPAchrome was measured at 475 nm in a microplate reader. The mushroom tyrosinase activities were calculated in the relation to the controls (samples without drug). The value IC_50_ (the concentration of a drug that inhibits a standard response by 50 %) was calculated on the basis of a dose-dependent inhibition curve, as described by Chung et al. [25].

### Superoxide dismutase assay

Superoxide dismutase (SOD) activity was measured using an assay kit (Cayman, MI, USA) according to the manufacturer’s instruction. One unit of SOD was defined as the amount of enzyme needed to produce 50 % dismutation of superoxide radical. SOD activity was expressed in U/mg protein.

### Catalase assay

Catalase (CAT) activity was measured using an assay kit (Cayman, MI, USA) according to the manufacturer’s instruction. One unit of CAT was defined as the amount of enzyme that causes the formation of 1.0 nmol of formaldehyde per minute at 25 °C. CAT activity was expressed in nmol/min/mg protein.

#### Glutathione peroxidase assay

Glutathione peroxidase (GPx) activity was measured using an assay kit (Cayman, MI, USA) according to the manufacturer’s instruction. One unit of GPx was defined as the amount of enzyme that catalyzes the oxidation of 1 nmol of NADPH per minute at 25 °C. GPx activity was expressed in nmol/min/mg protein.

### Statistical analysis

In all experiments, mean values of at least three separate experiments (*n* = 3) performed in triplicate ± standard error of the mean (S.E.M.) were calculated. The results were analyzed statistically using GraphPad Prism 6.01 Software. A value of *p* < 0.05 (*) or *p* < 0.01 (**), obtained with a Student’s *t* test by comparing the data with those for control (cells without drugs), was considered statistically significant.

## Results

### The effect of norfloxacin and moxifloxacin on cell viability

**Fig. 1:**
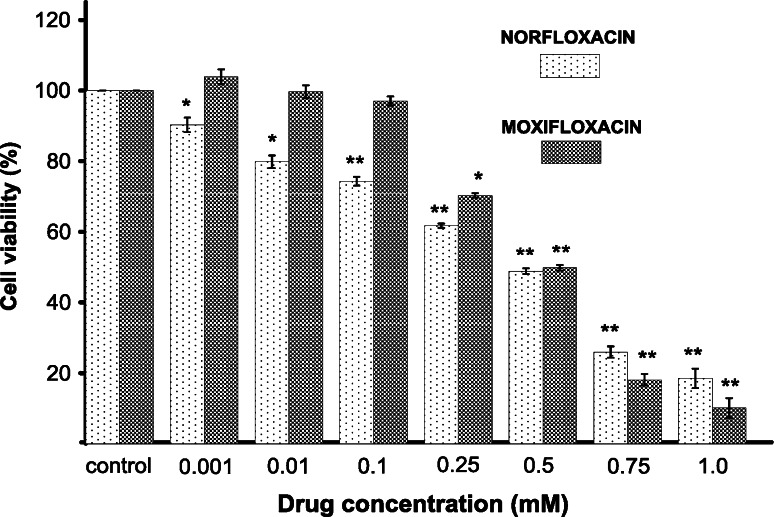
The effect of norfloxacin and moxifloxacin on viability of melanocytes. Cells were treated with various drugs concentrations (0.001–1.0 mM) and examined by the WST-1 assay. Data are expressed as  % of the controls. Mean values ± S.E.M. from three independent experiments (*n* = 3) performed in triplicate are presented. * *p* < 0.05 versus the control samples; ** *p* < 0.005 versus the control samples

Melanocytes were treated with norfloxacin or moxifloxacin in a range of concentrations from 0.001 to 1.0 mM for 24 h. The cell viability was determined by the WST-1 test assay. As shown in Fig. 1, norfloxacin induced an evident concentration-dependent loss in cell viability. Cells treated with 0.001, 0.01, 0.1, 0.25, 0.5, 0.75, and 1.0 of norfloxacin lost 9.8, 20.2, 25.7, 38.3, 51.2, 74.1, and 81.5 % in viability, respectively. In contrast to norfloxacin, moxifloxacin in concentrations from 0.001 mM to 0.1 mM did not influence the cell viability. After incubation of cells with 0.25, 0.5, 0.75, and 1.0 of moxifloxacin, the loss in cell viability was 29.7, 50.2, 89.1, and 89.9 %, respectively. For the both analyzed drugs, the value of EC_50_ (i.e., the amount of a drug that produces loss in cell viability by 50 %) was 0.5 mM.

### The effect of norfloxacin and moxifloxacin on melanization process

**Fig. 2:**
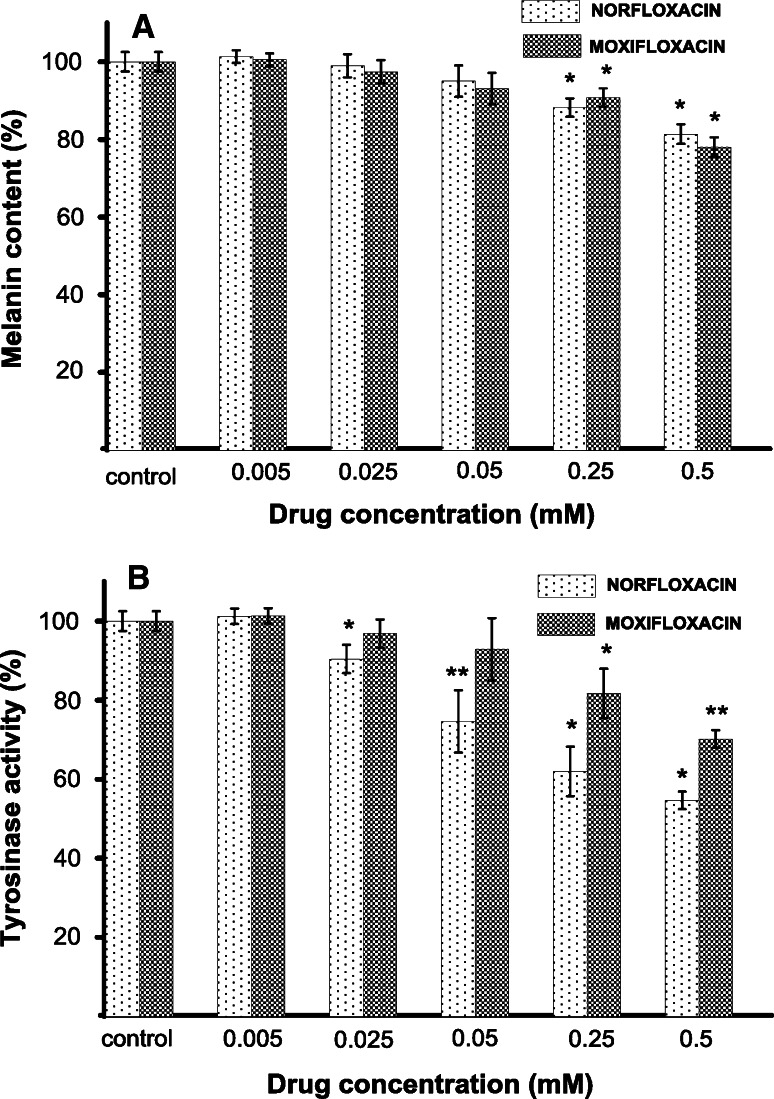
The effect of norfloxacin and moxifloxacin on melanin content (**a**) and tyrosinase activity (**b**) in melanocytes. Cells were cultured with norfloxacin or moxifloxacin in concentrations from 0.005 to 0.5 mM for 24 h. The melanin content and cellular tyrosinase activity were measured as described in “Materials and methods” section. Results are expressed as percentages of the controls. Data are mean ± S.E.M. of at least three independent experiments (*n* = 3) performed in triplicate. * *p* < 0.05 versus the control samples; ** *p* < 0.005 versus the control samples

The effectiveness of melanization process was estimated by measuring the melanin content and cellular tyrosinase activity in melanocytes treated with norfloxacin or moxifloxacin in concentrations from 0.005 mM to 0.5 mM, for 24 h. After determining a calibration curve, the melanin content per cell was estimated as 37.3 ± 0.6 to 28.3 ± 0.9 pg/cell and 36.2 ± 0.5 to 28.1 ± 3.04 pg/cell for melanocytes treated with norfloxacin and moxifloxacin, respectively, while the value determined for the control (cell without drug) was 36.6 ± 3.4 pg/cell. The obtained results, recalculated for culture (1 × 10^5^ cells), were finally expressed as a percentage of the controls (Fig. 2a). Both analyzed drugs in concentration of 0.005, 0.025, and 0.05 had no statistically significant effect on melanin content in melanocytes. In cells treated with norfloxacin or moxifloxacin at concentrations of 0.25 mM and 0.5 mM for 24 h, melanin production decreased by about 12 % and 19 % or 10 % and 22 %, respectively.

Tyrosinase activity in HEMa-LP cells treated with moxifloxacin decreased in a manner correlating well with the inhibitory effect on melanin production (Fig. 2b). After 24-h incubation tyrosinase activity was suppressed to 82 % at 0.25 mM and to 70 % at 0.5 mM when compared with the control. The use of moxifloxacin in lower concentrations (0.005, 0.025 and 0.05) had no statistically significant effect on the enzyme activity. In contrast to moxifloxacin, norfloxacin significantly decreased tyrosinase activity by 10, 25, 38, and 45 % at concentrations of 0.025, 0.05, 0.25, and 0.5, respectively. Only the lowest drug concentration (0.005 mM) had no effect on the enzyme activity.

The analyzed drugs significantly decreased mushroom tyrosinase activity (Table 1) in a concentration-dependent manner. The concentration of norfloxacin and moxifloxacin required for 50 % inhibition of mushroom tyrosinase activity (IC_50_) was 0.7 mM and 1.2 mM, respectively, what supports the cellular results indicating higher inhibitory effect of norfloxacin than moxifloxacin on this enzyme activity.

**Table 1:** The inhibitory effect of norfloxacin and moxifloxacin on mushroom tyrosinase activity

Analyzed drug	Inhibition ± S.E.M^a^ (%)	IC_50_^b^ (mM)
Norfloxacin 0.005 mM	77.3 ± 3.4	0.70
Norfloxacin 0.05 mM	67.7 ± 2.1	
Norfloxacin 0.5 mM	53.4 ± 2.3	
Norfloxacin 1.0 mM	44.7 ± 3.6	
Moxifloxacin 0.005 mM	76.6 ± 1.3	1.20
Moxifloxacin 0.05 mM	70.9 ± 1.7	
Moxifloxacin 0.5 mM	61.8 ± 3.1	
Moxifloxacin 1.0 mM	56.1 ± 2.5	

^a^Samples-contained phosphate buffer with different norfloxacin or moxifloxacin concentrations, mushroom tyrosinase (1,000 units), and L-DOPA solution (2 mg/ml). Tyrosinase activity was measured as described in “Materials and methods” section

^b^50 % inhibitory concentration

### The effect of norfloxacin and moxifloxacin on antioxidant enzymes activity

**Fig. 3:**
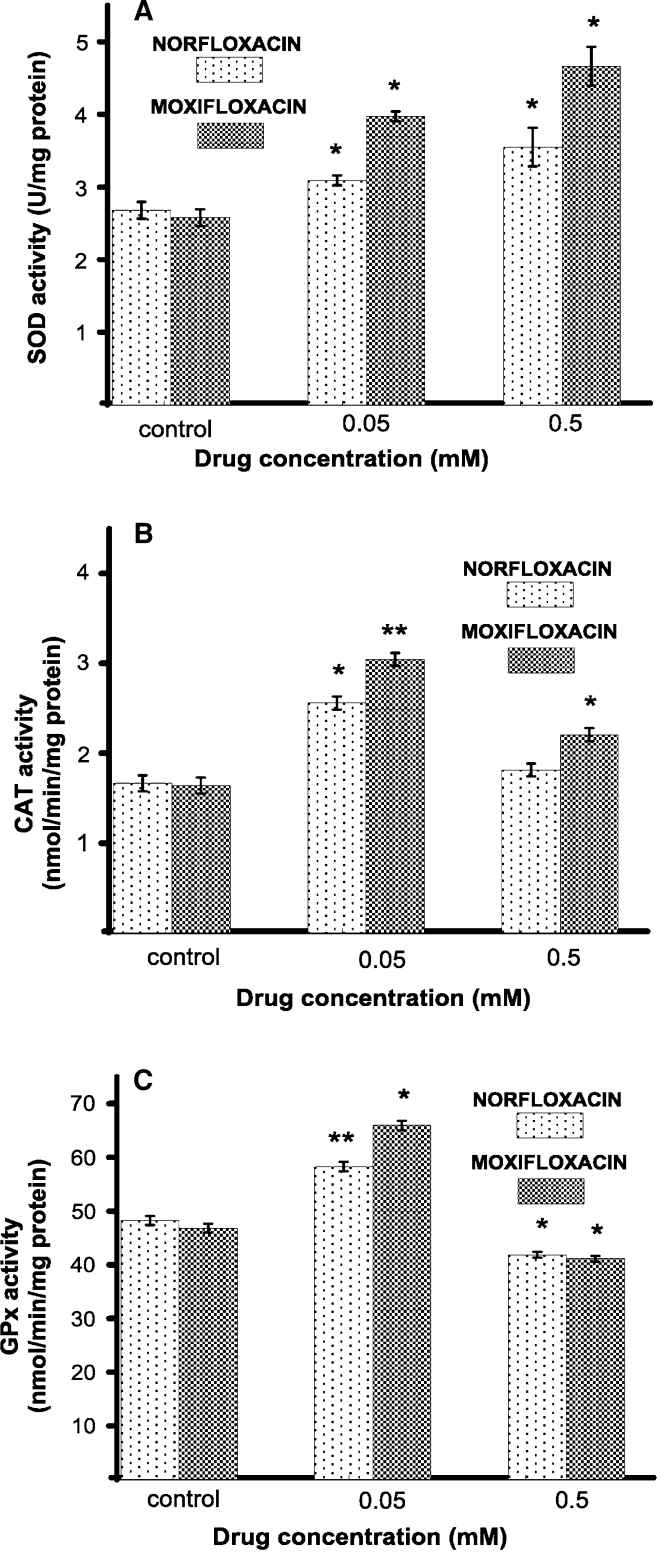
The superoxide dismutase (SOD) (**a**), catalase (CAT) (**b**), and glutathione peroxidase (GPx) (**c**) activity in HEMa-LP cells after 24-h incubation with 0.05 or 0.5 mM of norfloxacin and moxifloxacin. Data are mean ± S.E.M. of at least three independent experiments (*n* = 3) performed in triplicate. * *p* < 0.05 versus the control samples; ** *p* < 0.005 versus the control samples

To explain the effect of the tested antibiotics on reactive oxygen species metabolism, the activities of the antioxidative enzymes were characterized. Human melanocytes HEMa-LP were exposed to norfloxacin or moxifloxacin in concentrations of 0.05 mM and 0.5 mM (EC_50_) for 24 h. The first enzyme measured was SOD, i.e., the enzyme which catalyzes the formation of hydrogen peroxide from superoxide anion. Both analyzed drugs enhanced the SOD activity in a concentration-dependent manner (Fig. 3a). The treatment of cells with 0.05 mM and 0.5 mM of norfloxacin or moxifloxacin increased the SOD activity by 15 and 32 %, or by 54 and 81 %, respectively, as compared with the controls. CAT and GPx work together to catalyze the breakdown of hydrogen peroxide, produced by SOD, to water. The intracellular CAT activity was significantly increased by 54 or 86 % for cells treated with norfloxacin or moxifloxacin at 0.05 mM concentration. Norfloxacin in concentration of 0.5 mM (EC_50_) increased the CAT activity only by 9 %, whereas moxifloxacin increased the enzyme activity by 35 % (Fig. 3b). The activity of GPx increased by 20 or 40 % for norfloxacin or moxifloxacin in concentration of 0.05 mM and decreased by 13 or 12 % for cells treated with 0.5 mM (EC_50_), respectively, in comparison to control cells (Fig. 3c).

## Discussion

The fluoroquinolone antibiotics are used in the treatment of a wide range of bacterial infections, but are recognized as a group to be associated with drug-induced phototoxicity, especially directed to skin tissues [2, 3, 8, 10]. Among clinically developed fluoroquinolones phototoxicity ranks as follows: sparfloxacin ≥ lomefloxacin (both carrying a halogen in position 8) > ciprofloxacin ≥ norfloxacin > ofloxacin ≫ moxifloxacin [1, 2]. Although the phototoxicity of fluoroquinolones is well documented, the molecular mechanisms have not yet been precisely determined.

Many investigators have demonstrated the affinity of natural and synthetic melanins for various drugs by in vitro studies [18, 26, 27]. By inhibiting or significantly restricting drug access to cell receptors, melanins protect organism against undesirable drugs’ side effects. However, long-term exposure and slow release of drugs or their metabolites from bonds may increase the level of noxious substances stored on melanin, which may cause degeneration in the melanin-containing cells (e.g., in the skin and eye) and surrounding tissues [17, 18].

Previously, we have demonstrated that norfloxacin and moxifloxacin interact specifically with melanin, what may be a reason for these drugs accumulation in melanin-rich tissues [28, 29]. When comparing the binding parameters obtained for moxifloxacin [28] with those of norfloxacin, sparfloxacin [28], and lomefloxacin [20], it can be observed that the total number of binding sites in the melanin biopolymer for moxifloxacin was significantly lower than that for the other analyzed fluoroquinolones. Simultaneously, the moxifloxacin-melanin complexes were characterized by lower values of stability constants. The different affinity and capacity for binding of the studied fluoroquinolone derivatives to melanin may explain various toxic activities of these drugs on pigmented tissues in vivo.

Melanin is a biopolymer synthesized by melanocytes in discrete organelles— melanosomes [15, 30]. Therefore, in this study, we have used the culture of normal human epidermal melanocytes as an in vitro experimental model system. The study was conducted to elucidate the possible involvement of melanin and melanocytes in the mechanisms of fluoroquinolones-induced toxic effects. The melanin content and tyrosinase activity were chosen as the biomarkers for modulatory effect of norfloxacin and moxifloxacin on melanogenesis, whereas the SOD, CAT, and GPx activities as the markers for oxidative stress in normal human melanocytes HEMa-LP.

It was observed that moxifloxacin in concentrations from 0.001 to 0.1 mM did not have any significant effect on melanocytes viability. Higher drug concentrations (from 0.25 to 1.0 mM) resulted in the loss of cell viability in a concentration-dependent manner (Fig. 1). The use of norfloxacin caused higher reduction in cell viability when this drug was applied in lower concentrations. The value of EC_50_ for both analyzed drugs was determined to be 0.5 mM. For the same cell line, the value of EC_50_ established for other fluoroquinolones, ciprofloxacin and lomefloxacin, was 0.5 mM [19] and 0.75 mM [20], respectively, what indicates that norfloxacin and moxifloxacin are similarly cytotoxic.

Melanin is synthesized in the presence of tyrosinase, a copper-containing metalloglycoprotein which is attributed to possess multiple catalytic activities utilizing l-tyrosine, dihydroxyphenylalanine (L-DOPA), and 5,6-dihydroxyindole as substrates [31]. The rate limiting step in melanin synthesis is the hydroxylation reaction involving the conversion of the amino acid l-tyrosine to dihydroxyphenylalanine under the control of tyrosinase. DOPA is subsequently converted to a stable intermediate DOPAchrome, which undergoes a spontaneous decarboxylation to 5,6-dihydroxyindole (DHI) or an enzymatic tautomerization (by tyrosinase-related protein TRP2) to 5,6-dihydroxyindole-2-carboxylic acid (DHICA). Both of these products are then oxidized to quinones, which are polymerized to eumelanin. In the presence of sulphydryl compounds (cysteine or glutathione), the synthetic pathway is shifted toward the formation of pheomelanins. [15, 30–32].

Since tyrosinase is a major regulator of melanin synthesis, we have examined a direct effect of norfloxacin and moxifloxacin on the activity of this enzyme. Norfloxacin in concentrations of 0.025, 0.05, 0.25, and 0.5 suppressed the tyrosinase activity in melanocytes to 90, 75, 62, and 55 %, respectively (Fig. 2b). The inhibitory effect of moxifloxacin on cellular tyrosinase activity was observed only at higher drug concentrations (from 0.25 and 0.5 mM). Thus, norfloxacin demonstrates greater inhibitory effect on tyrosinase activity in melanocytes than moxifloxacin. We repeated the experiment with commonly used mushroom tyrosinase and obtained similar results (Table 1). Our findings indicate that an inhibitory effect of the studied antibiotics on melanogenesis is probably due to their direct inhibition of tyrosinase activity. The results of our prior studies [19, 20] and those obtained in this study reveal that moxifloxacin demonstrates the lowest inhibitory effect on cellular tyrosinase activity in comparison to norfloxacin, ciprofloxacin, or lomefloxacin.

The analysis of melanin formation in cells cultured in the presence or absence of a drug showed that norfloxacin and moxifloxacin in concentrations of 0.25 mM or 0.5 mM suppressed the melanin content to 88 and 81 %, or to 90 or 78 %, respectively (Fig. 2a). In comparison to ciprofloxacin [19] and lomefloxacin [20], the drugs analyzed in this study caused lower reduction in melanin content.

It has been suggested that free radicals generated after fluoroquinolones treatment play an important role in these antibiotics’ toxicity [3, 8, 33]. Several antioxidant enzymes, including superoxide dismutase (SOD), catalase (CAT), and glutathione peroxidase (GPx), are effective in removing destructive ROS. Insufficient activity of intracellular antioxidant enzymes can cause damage to cell structures. When unbalanced, it may lead to oxidation of polyunsaturated fatty acids in lipids, amino acids in proteins, and damage to DNA [34, 35].

Melanin is known to be a scavenger of free radicals and it has been suggested that it possesses the superoxide dismutase activity [36]. Moreover, this biopolymer acts as a biochemical dustbin, mopping up potentially toxic agents [18]. Such properties may be important for protecting the pigment cells as well as surrounding tissues from the natural toxins, xenobiotics, oxygen and free radicals, including ROS [14].

In the present study, it has been observed for the first time that norfloxacin and moxifloxacin cause significant changes in the activities of the antioxidant enzymes: SOD, CAT, and GPx in melanocytes. SOD protects cells by dismutating superoxide anion into the proradical hydrogen peroxide, which in turn is inactivated to oxygen and water by catalase or other H_2_O_2_-removing enzymes such as glutathione peroxidase [34]. In melanocytes, catalase is the main enzyme responsible for degrading hydrogen peroxide [37]. If H_2_O_2_ is not effectively cleared, level of reactive hydroxyl radicals may increase due to iron-catalyzed Fenton-type reactions [34]. Here presented a concentration-dependent increase in SOD activity after exposure of melanocytes to norfloxacin and moxifloxacin (Fig. 3a) might be the main reason for overproduction of the superoxide anion and subsequent formation of H_2_O_2_, what leads to the increase in CAT activity. Hsiao et al. [38] have shown that an other fluoroquinolone derivative, namely trovafloxacin, causes mitochondrial peroxynitrite stress in a mouse model leading to the disruption of mitochondrial enzymes and gene regulation. The observed in the present study lower increase of SOD activity in melanocytes after cells exposure to norfloxacin than to moxifloxacin may be caused by this drug higher ability to form reactive nitrogen species (RNS), such as peroxynitrite which may be generated in the presence of high level of superoxide anion leading to SOD inactivation [39]. Moreover, Martinez et al. [33] have proved the ability of norfloxacin to generate high amounts of superoxide anion. Treatment of cells with norfloxacin and moxifloxacin in concentration of 0.05 mM caused higher increase in CAT activity than in concentration of 0.5 mM (Fig. 3b). When comparing the both analyzed drugs, it can be observed that the increase of CAT activity in melanocytes exposed to norfloxacin is significantly lower than in cells exposed to moxifloxacin. Qin and Liu [40] have demonstrated that fluoroquinolones could bind into CAT central cavity what may cause the conformational changes of this enzyme as well as lead to inhibition of its molecular activity. It could be then suggested, that norfloxacin possesses higher ability for binding to CAT central cavity than moxifloxacin and therefore, in its presence higher reduction of this enzyme activity may be observed. This phenomenon indicates for higher depletion of antioxidant status in melanocytes over norfloxacin treatment as compared with moxifloxacin. In addition, the tested antibiotics in concentration of 0.5 mM decreased GPx activity (Fig. 3c). Thus, it may be assumed that alterations in SOD, CAT, and GPx activity play a critical role in these drugs toxicity as a result of redundant superoxide anion and H_2_O_2_ level that cannot be eliminated.

One has to take into consideration that norfloxacin and moxifloxacin concentrations found to have an inhibitory effect on melanogenesis and antioxidant defense system in normal human melanocytes are about 16-, 160-fold higher [41], and 8-, 80-fold higher [42] for norfloxacin and moxifloxacin, respectively, than the concentrations normally observed in vivo. However, we have previously demonstrated that norfloxacin [28] and moxifloxacin [29] form complexes with melanin, what may lead to the accumulation of these drugs in melanin reach tissues. Such accumulation increases the drugs concentrations and may induce some toxic effects on melanin-containing cells and surrounding tissues. Thus, it is possible that norfloxacin and moxifloxacin concentrations in melanocytes may be significantly higher than that in serum and therefore the reduction of melanin content, the inhibition of tyrosinase activity as well as the inhibition of antioxidant enzymes activities in the presence of these drugs could be observed.

The obtained results give a new insight into the mechanisms of fluoroquinolones toxicity directed to pigmented tissues. Moreover, the presented differences in modulation of biochemical processes in melanocytes may be an explanation for various phototoxic activities of the analyzed fluoroquinolone derivatives in vivo.
